# Incidence of Ventricular Arrhythmias and Sudden Cardiac Death with Cardiac Myosin Inhibitors in Hypertrophic Cardiomyopathy: A Meta-Analysis of Randomized Controlled Trials

**DOI:** 10.3390/jpm16030159

**Published:** 2026-03-13

**Authors:** Josip Katic, Tomislav Bulum, Josip Anđelo Borovac

**Affiliations:** 1 Department of Cardiovascular Diseases, University Hospital of Split, 21000 Split, Croatiajborovac@mefst.hr (J.A.B.); 2 Department of Diabetes and Endocrinology, Vuk Vrhovac University Clinic for Diabetes, Endocrinology and Metabolic Diseases, Merkur University Hospital, 10000 Zagreb, Croatia; 3 School of Medicine, University of Zagreb, Šalata 3, 10000 Zagreb, Croatia; 4 Department of Health Studies, University of Split, 21000 Split, Croatia; 5 Department of Pathophysiology, University of Split School of Medicine, 21000 Split, Croatia

**Keywords:** hypertrophic cardiomyopathy, cardiac myosin inhibitors, ventricular arrhythmias, sudden cardiac death, randomized controlled trials

## Abstract

**Background:** Hypertrophic cardiomyopathy (HCM) is associated with an elevated risk of ventricular arrhythmias and sudden cardiac death (SCD) despite contemporary therapy. Cardiac myosin inhibitors directly target sarcomeric hypercontractility and have demonstrated consistent symptomatic, hemodynamic, and structural benefits in randomized controlled trials (RCTs). However, their effects on malignant ventricular arrhythmias and SCD remain uncertain. This meta-analysis aimed to evaluate the incidence of ventricular arrhythmias and SCD with cardiac myosin inhibitor therapy in HCM. **Methods:** We conducted a meta-analysis of RCTs evaluating mavacamten or aficamten in patients with HCM. PubMed was systematically searched through September 2025. Eligible trials randomized myosin inhibitors versus control and reported ventricular tachycardia (VT), ventricular fibrillation (VF), or SCD. **Results:** Seven RCTs including 1519 patients (779 myosin inhibitor; 740 control) were analyzed. Eight composite VT/VF/SCD events (1.03%) occurred in the treatment group compared with twelve (1.62%) in controls. Time-standardized incidence rates were 1.48 versus 2.36 per 100 patient-years, respectively. The pooled RR was 0.69 (95% CI 0.27–1.74; I^2^ = 0%), indicating no statistically significant difference. Sensitivity analyses yielded concordant results despite low event counts. **Conclusions:** No statistically significant increase in ventricular arrhythmia or SCD risk was observed. However, limited events and short follow-up preclude firm conclusions regarding the arrhythmic safety of myosin inhibitors.

## 1. Introduction

Hypertrophic cardiomyopathy (HCM) is the most common inherited cardiomyopathy and remains a major cause of heart failure symptoms, atrial and ventricular arrhythmias, and sudden cardiac death (SCD), particularly in younger individuals and selected high-risk phenotypes [1]. According to the 2023 European Society of Cardiology (ESC) guidelines for the management of cardiomyopathies, risk stratification in HCM requires an integrated assessment of clinical characteristics, imaging findings, family history, and arrhythmic markers, with prevention of SCD remaining a central therapeutic objective through individualized implantable cardioverter-defibrillators (ICDs) considered for selected patients [1]. Clinically, HCM is broadly divided into obstructive and non-obstructive phenotypes [2]. Obstructive HCM, characterized by dynamic left ventricular outflow tract obstruction, is present in most symptomatic patients and is typically associated with exertional dyspnea, chest pain, syncope, and reduced exercise capacity [3]. In contrast, non-obstructive HCM lacks a significant outflow tract gradient but may still be associated with substantial symptoms, impaired diastolic function, myocardial fibrosis, and arrhythmic risk [4]. Emerging evidence also suggests important sex-related differences in HCM. Female patients are often diagnosed at an older age and present with more advanced symptoms, higher prevalence of obstructive physiology, and more pronounced diastolic dysfunction compared with men, highlighting potential disparities in disease recognition and progression [5]. Contemporary ESC guidance emphasizes an individualized management strategy in HCM, integrating symptom severity, obstruction status, myocardial substrate, family history, and arrhythmic risk markers to guide decisions regarding pharmacotherapy, septal reduction therapy, and implantable cardioverter-defibrillator implantation [1]. Although conventional pharmacological treatment with beta-blockers, non-dihydropyridine calcium-channel blockers, and disopyramide remains the first-line therapy for symptomatic obstructive HCM, these agents do not directly target the underlying sarcomeric hypercontractility that characterizes the disease substrate [2,3,4]. Cardiac myosin inhibitors, agents that directly modulate sarcomeric contractility, target the hypercontractile substrate and have reshaped HCM management [1,2,3]. Multiple randomized controlled trials (RCTs) have assessed their efficacy and safety across the HCM spectrum, showing consistent hemodynamic and symptomatic benefits [6]. However, evidence on ventricular arrhythmias and SCD is limited and heterogeneous; because individual trials are underpowered for these rare outcomes, a systematic analysis is needed.

## 2. Materials and Methods

We conducted a meta-analysis of RCTs of myosin inhibitors, mavacamten and aficamten, focused on ventricular tachycardia (VT), ventricular fibrillation (VF), and SCD.

This systematic review and meta-analysis were conducted and reported in accordance with the PRISMA 2020 statement [7]. This meta-analysis was not registered a priori in PROSPERO prior to study initiation.

Our objectives were to estimate the pooled effect, examine heterogeneity, and test robustness via sensitivity analyses. We systematically searched PubMed to September 2025 using “myosin inhibitor,” “mavacamten,” “aficamten,” and “hypertrophic cardiomyopathy”. Reference lists of eligible articles and relevant reviews were additionally screened to identify further studies. Studies were eligible if they: (1) were randomized controlled trials; (2) enrolled adult patients with HCM (obstructive or nonobstructive); (3) compared mavacamten or aficamten with placebo or active control; and (4) reported data on ventricular tachycardia (VT), ventricular fibrillation (VF), and/or sudden cardiac death (SCD) (including within safety/adverse event reporting). No restrictions were applied to the trial phase, follow-up duration, background therapy, or publication status within PubMed indexing. Ventricular arrhythmia and sudden cardiac death events were extracted as reported in the original randomized trials, based on study-specific safety definitions and adjudication processes, which were not fully harmonized across studies.

The literature search was limited to a single database, which may have resulted in the omission of unpublished or non-indexed studies. Sixteen records were identified and assessed in full text; of these, seven RCTs met the inclusion criteria. Title/abstract screening and data extraction were performed independently by two investigators, with discrepancies resolved by consensus.

Risk of bias for each included randomized trial was evaluated using the Cochrane Risk of Bias 2 (RoB 2) tool at the outcome level, focusing on the composite safety endpoint of ventricular tachycardia/ventricular fibrillation/sudden cardiac death (VT/VF/SCD) [8]. Judgments were made across the five RoB 2 domains: (D1) randomization process, (D2) deviations from intended interventions, (D3) missing outcome data, (D4) measurement of the outcome, and (D5) selection of the reported result, and summarized as low risk, some concerns, or high risk. Because malignant ventricular arrhythmic events were rare and were typically captured through adverse event safety reporting frameworks rather than as prespecified efficacy endpoints, particular attention was paid to (i) blinding and adherence (D2), (ii) completeness of follow-up and safety reporting.

Finally, we assessed the certainty of evidence for the primary safety endpoint (composite VT/VF/SCD) using the GRADE approach across the domains of risk of bias, inconsistency, indirectness, imprecision, and publication bias [9]. Evidence from randomized trials was initially rated as high certainty and was downgraded as appropriate. A Summary of Findings table was generated, reporting relative and absolute effects and the overall certainty rating.

## 3. Results

**Figure 1:**
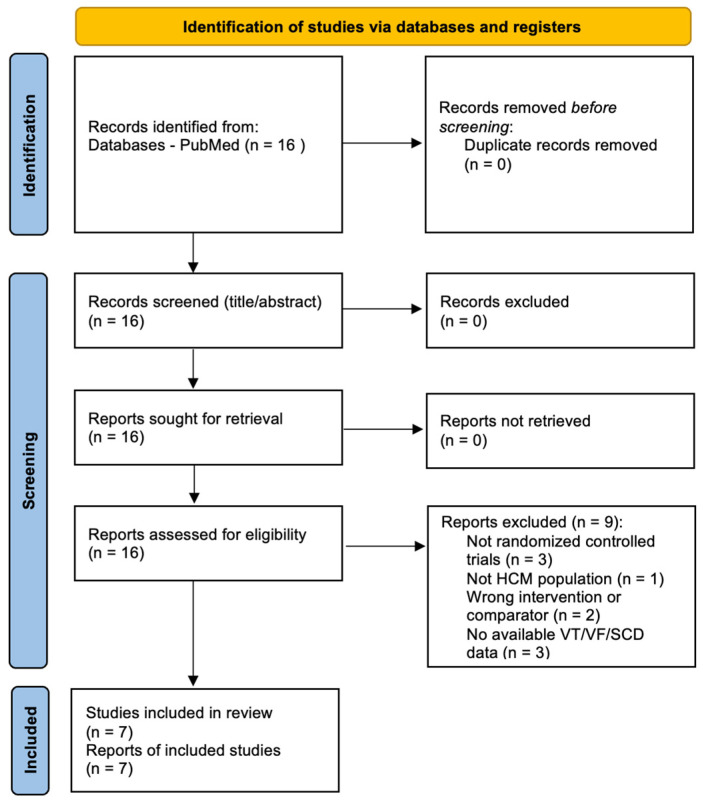
PRISMA 2020 study inclusion flowchart.

The PubMed search identified 16 records, all of which were retrieved for full-text assessment. After full-text review, 7 randomized controlled trials met the inclusion criteria and were included in the quantitative synthesis, while 9 reports were excluded (reasons: non-randomized design, non-HCM population, no eligible comparator, or no extractable VT/VF/SCD data). The PRISMA 2020 flow of study selection is presented in Figure 1.

Seven RCTs comprising 1519 participants (779 assigned to a myosin inhibitor and 740 to control) were included [10,11,12,13,14,15,16]. Trials evaluated mavacamten or aficamten versus placebo or active control, with follow-up durations ranging from short phase-2 exposure to 48-week outcomes in phase-3 programs.

**Figure 2:**
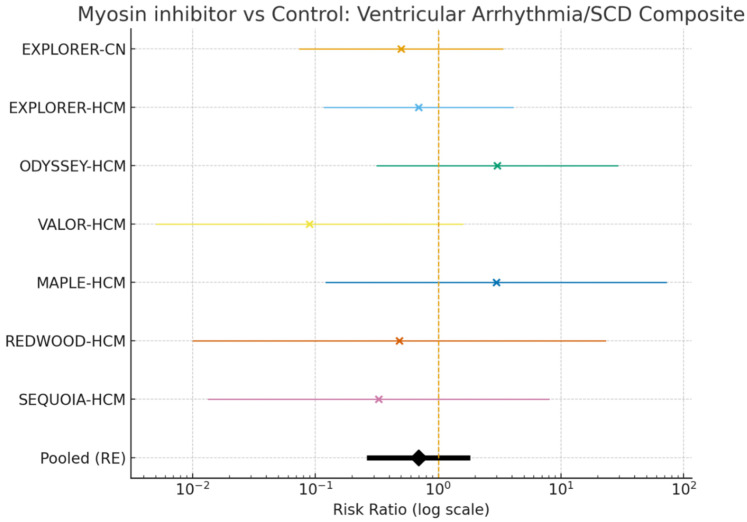
Forest plot of randomized trials illustrating the composite risk of ventricular arrhythmias/sudden cardiac death with cardiac myosin inhibitors versus control.

Across included trials, 8 composite VT/VF/SCD events occurred in the myosin inhibitor groups (1.03%) and 12 events in controls (1.62%). The pooled estimate showed no statistically significant difference in VT/VF/SCD with myosin inhibitors versus control (RR 0.69, 95% CI 0.27–1.74; I^2^ = 0%), although the point estimate favored myosin inhibition. Time-standardized incidence rates were 1.48 versus 2.36 events per 100 patient-years in the myosin inhibitor and control arms, respectively (Figure 2).

The baseline and main outcome data with absolute numbers and interventions explained for each included trial are shown in Table 1.

Across all RCTs, the incidence of the composite of SCD/VT/VF was approximately 1.48 per 100 patient-years in myosin-inhibitor arms (8 events over ≈541.2 patient-years) versus 2.36 per 100 patient-years in control arms (12 events over ≈508.8 patient-years) (Figure 2).

These rates mirror the direction of the pooled RR and provide an intuitive metric for clinical interpretation. The primary effect measure was the risk ratio (RR), estimated using the inverse-variance method with a 0.5 continuity correction applied when any cell contained zero events. A DerSimonian–Laird random-effects model was used, which was equivalent to the fixed-effects model in this case because between-study variance was null (τ^2^ = 0). The pooled RR was 0.69 (95% CI 0.27–1.74); I^2^ = 0%; τ^2^ = 0, and low heterogeneity was observed. The direction of effect favored myosin inhibitors, but the result was not statistically significant. The confidence interval encompassed both a potentially important benefit and harm. In the sensitivity analysis, using the risk difference (RD) under a random-effects model (excluding double-zero studies), the result was RD = −0.0019 (95% CI −0.0160 to 0.0121); I^2^ ≈ 45%. An additional sensitivity analysis using the Peto odds ratio (OR) yielded Peto OR = 1.00 (95% CI 0.94–1.06). It should be noted that this method may bias estimates toward the null in the presence of unbalanced study arms or single-zero trials. From trials with publicly available baseline ICD data in the primary report, ICD prevalence ranged from 12.1% to 22.7% across study arms, with an overall mean of ~17.7% when pooling across the reporting arms.

**Figure 3:**
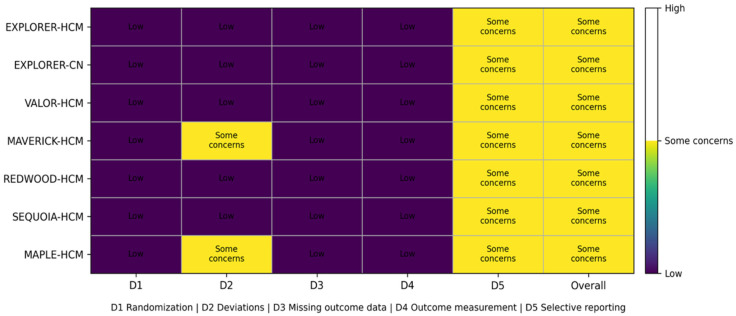
Risk of Bias 2 (RoB 2) assessment of included randomized trials.

Overall, RoB 2 assessment suggested low risk of bias for domains related to randomization (D1), missing outcome data (D3), and outcome measurement (D4) across all included trials (Figure 3). Some concerns were identified in D2 (deviations from intended interventions) for MAVERICK-HCM and MAPLE-HCM, reflecting design and conduct features that may increase susceptibility to performance-related deviations relative to fully blinded, placebo-controlled trials. In addition, all trials were rated as some concerns for D5 (selection of the reported result) for the VT/VF/SCD composite because malignant arrhythmic events were infrequent and generally reported as part of safety adverse-event summaries, with limited publicly available detail on prespecification, harmonized definitions, and adjudication of arrhythmic outcomes for this specific endpoint. Consequently, the overall RoB 2 judgment was “some concerns” for each study, driven primarily by uncertainty around selective reporting for rare safety events rather than evidence of systematic flaws in randomization, follow-up completeness, or outcome ascertainment.

To maintain scrutiny, we performed a GRADE assessment of selected studies with respect to the designated outcome. The certainty of evidence for the VT/VF/SCD composite was rated very low (Supplementary Figure S1). Downgrading was driven by very serious imprecision due to rare events and wide confidence intervals, indirectness related to non-harmonized and largely safety-report-derived arrhythmic outcome ascertainment, and risk-of-bias concerns primarily related to potential selective reporting of rare safety events. The completed PRISMA 2020 checklist is provided (Supplementary Table S1).

## 4. Discussion

In this ad hoc meta-analysis of RCTs evaluating cardiac myosin inhibitors in HCM, no excess risk of ventricular arrhythmias or SCD was observed compared with the control arm. Although the point estimate favored myosin inhibitors, statistical significance was not reached, and the confidence intervals encompassed both potential benefits and harms. These findings are consistent with the overall safety data reported in individual trials, in which ventricular tachyarrhythmias were rare events and occurred sporadically in both treatment and control groups.

The review by Pagel et al. emphasized that the HCM hypercontractile state arises from enhanced myosin–actin crossbridge formation and destabilization of the energy-conserving super-relaxed state, leading to chronic energetic stress, hypertrophy, and myocardial fibrosis, key contributors to arrhythmogenesis. By selectively reducing myosin ATPase activity and stabilizing the super-relaxed state, mavacamten and aficamten decrease wall stress and oxygen demand, mechanisms that could theoretically reduce arrhythmic triggers rather than exacerbate them [6]. Indeed, across pivotal RCTs, no signal of proarrhythmic effect was reported; ventricular arrhythmias and SCD events were extremely infrequent. Importantly, both mavacamten and aficamten produced favorable cardiac remodeling, reductions in left ventricular outflow tract gradients, wall thickness, and NT-proBNP [1,2,3]. Such structural and hemodynamic improvements may contribute to long-term electrical stability. Available randomized data suggest that treatment benefits are more consistent in obstructive HCM, where a reduction in dynamic outflow tract gradient translates into symptomatic and functional improvement, whereas in non-obstructive HCM, the clinical response appears less pronounced [17]. That likely reflects greater pathophysiological heterogeneity and a larger contribution of diastolic dysfunction, myocardial fibrosis, and microvascular abnormalities [18]. Similarly, differences in sex, age at presentation, baseline fibrosis burden, and pre-existing arrhythmic substrate may further influence both therapeutic response and long-term safety, underscoring the need for individualized interpretation of emerging pharmacological data [19].

In interpreting the arrhythmic safety signal of cardiac myosin inhibitors, our RoB 2 assessment suggests that the included RCTs were generally robust with low risk of bias for the randomization process (D1), missing outcome data (D3), and outcome measurement (D4) for VT/VF/SCD, supporting the internal validity of the underlying trial conduct [8]. The principal limitation identified was “some concerns” for the selection of the reported result (D5) across trials, driven by the fact that malignant ventricular arrhythmias and SCD were rare and typically captured within adverse-event safety reporting frameworks rather than as uniformly prespecified, centrally adjudicated outcomes with harmonized definitions [7,8]. In two trials (MAVERICK-HCM and MAPLE-HCM), some concerns in deviations from intended interventions (D2) further highlight the possibility that treatment adherence, co-interventions, or differential follow-up intensity could influence rare safety event ascertainment, although the direction and magnitude of any resulting biases are difficult to predict [8]. Collectively, these considerations imply that the absence of an apparent proarrhythmic signal in pooled RCT data is reassuring, but confidence remains constrained by imprecision (very low event counts) and outcome-level reporting uncertainty; longer-term studies and registries incorporating systematic rhythm monitoring (including ICD interrogation data where applicable) and prespecified arrhythmia endpoints will be important to definitively characterize long-term arrhythmic safety [7,20].

There are some notable limitations of our work. This meta-analysis has several important limitations. First, the study was not prospectively registered in PROSPERO, which may reduce transparency regarding predefined methods and outcomes. Second, the literature search was restricted to a single database (PubMed), potentially leading to omission of unpublished, non-indexed, or gray literature studies. Because the pooled analysis remains limited by very low event counts and inadequate statistical power, the absence of a significant signal should also be interpreted as reflecting the limited inferential capacity of the individual randomized trials, which were likewise not designed to provide definitive conclusions regarding arrhythmic safety. Accordingly, our findings should be interpreted primarily as a quantitative summary of currently available randomized evidence rather than as definitive evidence of arrhythmic safety.

Based on the observed event rates in the included studies (approximately 1.0% vs. 1.6%), a clinically robust assessment of safety would likely require substantially larger datasets than currently available. A rough power estimate suggests that detecting a difference in this magnitude with conventional statistical assumptions (80% power, two-sided alpha 0.05) would require approximately 11,000–12,000 participants overall, corresponding to well over 150 adjudicated VT/VF/SCD events, which is far beyond the cumulative event burden of currently available randomized trials. Fourth, follow-up duration across included trials was relatively short (10–48 weeks), precluding assessment of long-term arrhythmic risk and sudden cardiac death. Fifth, definitions and ascertainment of ventricular arrhythmias were not fully harmonized across trials, and events were largely reported within adverse event safety frameworks rather than as uniformly prespecified and centrally adjudicated arrhythmic endpoints. Furthermore, clinical heterogeneity existed across studies, including differences in HCM phenotype (obstructive vs. non-obstructive), background therapy, trial phase, and baseline ICD prevalence. Finally, the small number of included trials limited formal assessment of publication bias. Longer follow-up and studies with prespecified arrhythmia endpoints (including systematic rhythm monitoring/ICD interrogation where applicable) are required in the future to demonstrate arrhythmic safety.

## 5. Conclusions

In conclusion, in this meta-analysis of RCTs evaluating cardiac myosin inhibitors in HCM, no statistically significant increase in ventricular arrhythmias or SCD was observed. However, given the very low number of events, the analysis is underpowered, and firm conclusions regarding the arrhythmic safety of these drugs cannot be drawn. Definitive appraisal of SCD risk and arrhythmic burden will require longer, device-augmented monitoring and adequately powered trials across HCM phenotypes. While hemodynamic/structural benefits are consistent, long-term electrophysiological safety remains to be confirmed.

## Figures and Tables

**Table 1:** Baseline and main outcome data of included randomized controlled trials of myosin inhibitors in HCM.

Trial Name	Authors	PMID	N of Patients (Exp/Control)	Intervention (Dose, Schedule, Duration)	Main Outcomes (Primary and Key Secondary with Effect Sizes, 95% CI)
EXPLORER-HCM (Lancet 2020) [16]	Olivotto, I.; Oreziak, A.; Barriales-Villa, R.; Abraham, T.P.; Masri, A.; Garcia-Pavia, P.; et al.	32871100	251 (Mava 123/Placebo 128)	Mavacamten start 5 mg daily, titrated; 30 weeks; vs. placebo; background therapy allowed.	Primary composite: 37% vs. 17%; Δ = +19.4% (95% CI 8.7–30.1), *p* = 0.0005. Secondary: Post-exercise LVOT gradient Δ −36 mmHg (95% CI −43.2 to −28.1), *p* < 0.0001; pVO_2_ Δ +1.4 mL/kg/min (95% CI 0.6–2.1), *p* = 0.0006; KCCQ-CSS Δ +9.1 (95% CI 5.5–12.7), *p* < 0.0001.
VALOR-HCM (JACC 2022) [15]	Desai, M.Y.; Owens, A.; Geske, J.B.; Wolski, K.; Naidu, S.S.; Smedira, N.G.; et al.	35798455	112 (Mava 56/Placebo 56)	Mavacamten 5–15 mg daily, titrated to LVOT gradient and LVEF; 16 weeks; vs. placebo; maximally tolerated background therapy.	Primary: 17.9% vs. 76.8%; absolute difference −58.9% (95% CI −73.9 to −44.0), *p* < 0.001. Secondary: Post-exercise LVOT gradient Δ −37.2 mmHg; ≥1 NYHA class improvement Δ +41.1%; NT-proBNP GMR 0.33 and hs-cTnI GMR 0.53; all *p* < 0.001.
EXPLORER-CN (JAMA Cardiol 2023) [14]	Tian, Z.; Li, L.; Li, X.; Wang, J.; Zhang, Q.; Li, Z.; et al.	37639259	81 (Mava 54/Placebo 27)	Mavacamten start 2.5 mg daily (CYP2C19-guided titration); 30 weeks; vs. placebo.	Primary: LSM Δ −70.3 mmHg (95% CI −89.6 to −50.9), 1-sided *p* < 0.001. Resting gradient LSM Δ −55.0 mmHg (95% CI −69.1 to −40.9). NYHA improvement: 59.3% vs. 14.8%. KCCQ-CSS LSM Δ +10.2 (95% CI 4.4–16.1).
SEQUOIA-HCM (NEJM 2024) [13]	Maron, M.S.; Masri, A.; Nassif, M.E.; Barriales-Villa, R.; Arad, M.; Cardim, N.; et al.	38739079	282 (Afica 142/Placebo 140)	Aficamten start 5 mg (up-titrate to 20 mg q2wk by echo); 24 weeks; vs. placebo; background therapy permitted.	Primary: LSM Δ +1.7 mL/kg/min (95% CI 1.0–2.4), *p* < 0.001. Secondary: all significant—KCCQ-CSS; NYHA class improvement; Valsalva gradient reduction; % with Valsalva gradient <30 mmHg; reduced SRT eligibility.
MAPLE-HCM (NEJM 2025) [12]	Garcia-Pavia, P.; Maron, M.S.; Masri, A.; Merkely, B.; Nassif, M.E.; Peña-Peña, M.L.; et al.	40888697	175 (Afica 88/Metoprolol 87)	Aficamten 5–20 mg daily vs. metoprolol 50–200 mg daily (double-dummy); 24 weeks; monotherapy.	Primary: LSM Δ +2.3 mL/kg/min (95% CI 1.5–3.1), *p* < 0.001 (favoring aficamten). Secondary: greater improvement in NYHA class, KCCQ-CSS, Valsalva gradient, NT-proBNP, and LAVI (no sig. Δ LVMI).
ODYSSEY-HCM (NEJM 2025) [11]	Desai, M.Y.; Owens, A.T.; Abraham, T.P.; Olivotto, I.; Garcia-Pavia, P.; Lopes, R.D.; et al.	40888717	580 (Mava 289/Placebo 291)	Mavacamten start 5 mg up to 15 mg; 48 weeks; vs. placebo; nonobstructive HCM.	Co-primary: Δ pVO_2_ +0.47 mL/kg/min (95% CI −0.03 to 0.98), *p* = 0.07; Δ KCCQ-CSS +2.7 (95% CI −0.1 to 5.6), *p* = 0.06—both NS. EF reductions and more regimen interruptions with mavacamten.
REDWOOD-HCM (JACC 2023, Phase 2) [10]	Maron, M.S.; Masri, A.; Choudhury, L.; Olivotto, I.; Saberi, S.; Wang, A.; et al.	36599608	41 (Afica 28/Placebo 13; two cohorts)	Aficamten randomized 2:1 vs. placebo; titrated by gradient/LVEF; 10 weeks + 2-week washout.	Resting gradient reduction vs. placebo: −40 ± 27 (*p* = 0.0003) and −43 ± 37 mmHg (*p* = 0.0004). Valsalva: −36 ± 27 (*p* = 0.001) and −53 ± 44 mmHg (*p* < 0.0001). NT-proBNP 62% vs. placebo (*p* = 0.0002).

## Data Availability

The data presented in this study are available on request from the corresponding author.

## Supplementary Materials

The following supporting information can be downloaded at: https://www.mdpi.com/article/10.3390/jpm16030159/s1, Figure S1: Summary of Findings (GRADE); Table S1: PRISMA 2020 checklist.
