# Supplementary figures and images for: Incidence of Ventricular Arrhythmias and Sudden Cardiac Death with Cardiac Myosin Inhibitors in Hypertrophic Cardiomyopathy: A Meta-Analysis of Randomized Controlled Trials
Source: J Pers Med. 2026 Mar 13;16(3):159. doi: 10.3390/jpm16030159 (PMC13028237; doi:10.3390/jpm16030159)

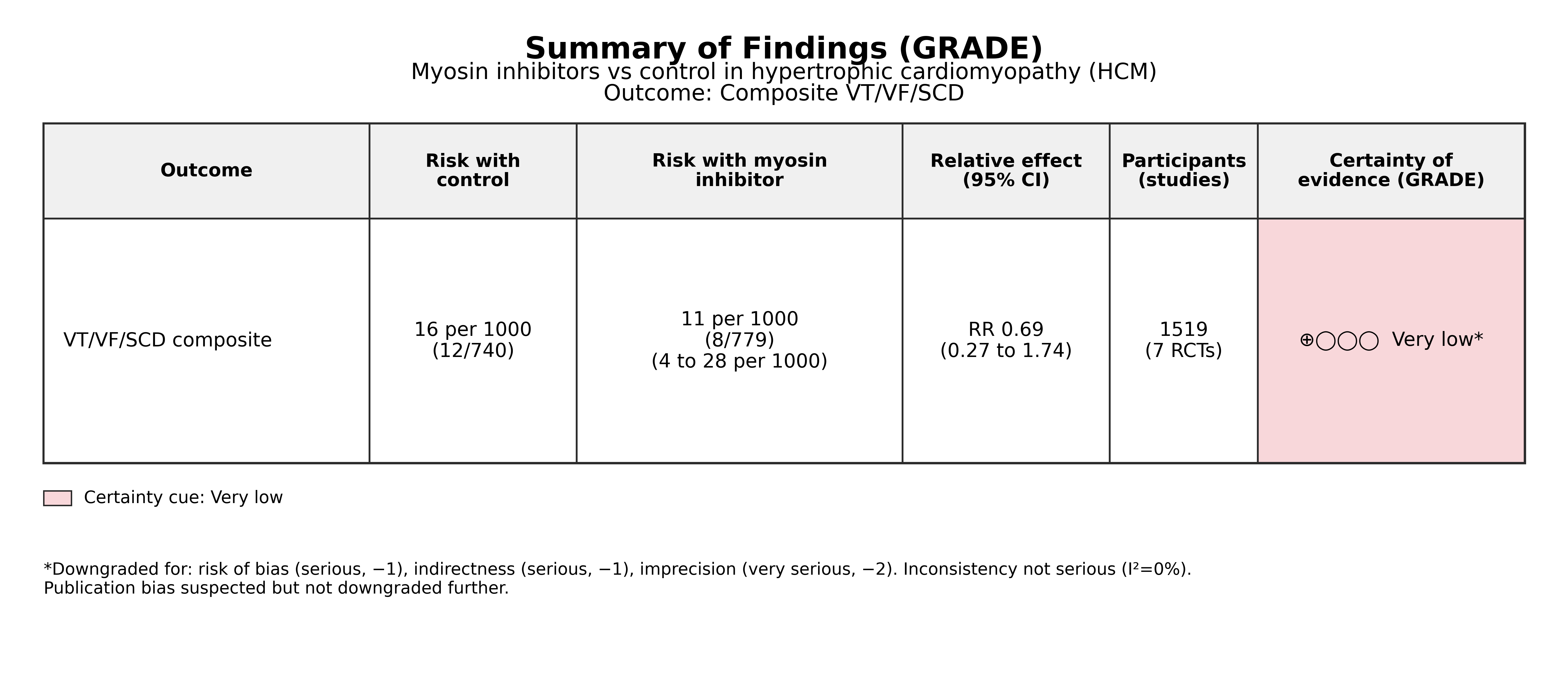

Supplement: Supplementary file 1 [file jpm-16-00159-s001.zip › jpm-4173688-Figure S1.png]
